# Atomically‐Precise Texturing of Hexagonal Boron Nitride Nanostripes

**DOI:** 10.1002/advs.202101455

**Published:** 2021-07-22

**Authors:** Khadiza Ali, Laura Fernández, Mohammad A. Kherelden, Anna A. Makarova, Igor Píš, Federica Bondino, James Lawrence, Dimas G. de Oteyza, Dmitry Yu. Usachov, Denis V. Vyalikh, F. Javier García de Abajo, Zakaria M. Abd El‐Fattah, J. Enrique Ortega, Frederik Schiller

**Affiliations:** ^1^ Centro de Física de Materiales CSIC/UPV‐EHU‐Materials Physics Center San Sebastián E‐20018 Spain; ^2^ Universidad del País Vasco Dpto. Física Aplicada San Sebastián E‐20018 Spain; ^3^ Physics Dept. Faculty of Science Al‐Azhar University Nasr City Cairo E‐11884 Egypt; ^4^ Freie Universität Berlin Berlin 14195 Germany; ^5^ IOM‐CNR Laboratorio TASC Trieste I‐34149 Italy; ^6^ Elettra ‐ Sincrotrone Trieste S.C.p.A. Trieste I‐34149 Italy; ^7^ Donostia International Physics Center San Sebastián E‐20018 Spain; ^8^ St. Petersburg State University St. Petersburg 199034 Russia; ^9^ Ikerbasque Basque Foundation for Science Basque Country Bilbao 48013 Spain; ^10^ ICFO‐Institut de Ciencies Fotoniques The Barcelona Institute of Science and Technology Barcelona 08860 Spain; ^11^ ICREA‐Institució Catalana de Recerca i Estudis Avançats Passeig Lluís Companys 23 Barcelona 08010 Spain

**Keywords:** boron nitride nanostripes, photoemission, scanning tunneling microscopy, uniaxial electronic bands

## Abstract

Monolayer hexagonal boron nitride (hBN) is attracting considerable attention because of its potential applications in areas such as nano‐ and opto‐electronics, quantum optics and nanomagnetism. However, the implementation of such functional hBN demands precise lateral nanostructuration and integration with other two‐dimensional materials, and hence, novel routes of synthesis beyond exfoliation. Here, a disruptive approach is demonstrated, namely, imprinting the lateral pattern of an atomically stepped one‐dimensional template into a hBN monolayer. Specifically, hBN is epitaxially grown on vicinal Rhodium (Rh) surfaces using a Rh curved crystal for a systematic exploration, which produces a periodically textured, nanostriped hBN carpet that coats Rh(111)‐oriented terraces and lattice‐matched Rh(337) facets with tunable width. The electronic structure reveals a nanoscale periodic modulation of the hBN atomic potential that leads to an effective lateral semiconductor multi‐stripe. The potential of such atomically thin hBN heterostructure for future applications is discussed.

## Graphical abstract

Vapor growth of borazine on a curved Rh surface leads to continuous hexagonal boron nitride (hBN) monolayer coating, under which nanomesh‐quantized Rh(111) terraces and stepped Rh(337) facets segregate, The latter reveals a perfect hBN/Rh interface registry, with all N atoms anchoring at step on‐top positions. This imprints a strong uniaxial anisotropy in the electronic band structure of the hBN monolayer.

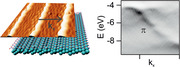

## 1 Introduction

Hexagonal boron nitride (hBN) is an attractive two‐dimensional (2D) material for high performance electronics and photonics applications. It competes as insulating substrate with SiO_2_ or Al_2_O_3_, and it is also promising as protective coating for metallic films that support intense 2D plasmons,^[^ ^1^, ^2^ ^]^ as a single photon emitter with deep UV lasing,^[^ ^3^ ^]^ or as a superconductor through suitable doping.^[^ ^4^ ^]^ Although hBN flakes may be obtained by mechanical exfoliation of bulk crystals, a single hBN monolayer can be readily synthesized on metal ‐surfaces,^[^ ^5^ ^]^ leading to structurally and chemically robust substrates that frequently exhibit nanoscale patterns. This makes hBN‐covered metals excellent platforms to achieve functional interfaces with atoms, molecules, and aggregates, as well as to develop hybrid 2D materials, such as twisted van der Waals stacks or 2D heterostructures.^[^ ^6^, ^7^, ^8^ ^]^ The latter hold a great potential for atomically thin circuitry, such as superstructures formed with isostructural graphene,^[^ ^9^, ^10^, ^11^, ^12^, ^13^ ^]^ which are optimal to engineer gaps and doping, as well as to tune and enhance spin scattering. However, exploiting fine hBN‐based nanostructures requires structural quality down to the atomic scale, which lies beyond current lithographic capabilities. The bottom‐up vapor growth is the alternative, which also works for 2D hybrids,^[^ ^14^ ^]^ although general procedures to control shape, size, and spatial order of surface phases are still lacking.

**Figure 1:**
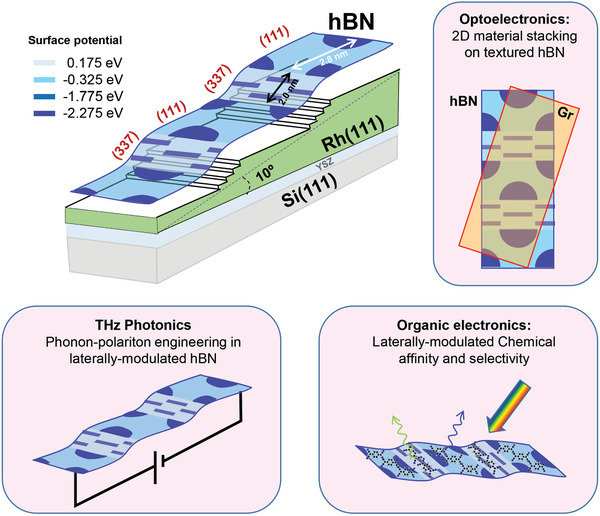
Lateral nanopatterning of hBN through epitaxial growth. We explore this concept by studying vicinal hBN/Rh interfaces. CVD growth of hBN induces periodic (111)/(337) faceting of the Rh substrate. The hBN monolayer uniformly coats the faceted substrate, defining an effective lateral hBN/hBN heterointerface with periodic surface potential texturing. Using commercial Rh(111) films^[^ ^17^, ^18^ ^]^ one could exploit such chemical and electronic modulation of hBN to explore phonon–polariton THz excitations, selective growth of optically‐active molecules, and vertical stacking with other 2D materials.

Here, we demonstrate the bottom‐up synthesis of nanostriped hBN heterostructures with atomically sharp interfaces. We follow the standard chemical vapor deposition (CVD) growth route, using Rhodium (Rh) vicinal surfaces as one‐dimensional (1D) templates. We show that the excellent registry with the Rh(337) plane triggers periodic (111)/(337) faceting of the substrate, as sketched in **Figure** **1**. The hBN monolayer carpet‐coats the faceted substrate, but the distinct symmetry and atomic interaction at each hBN/Rh phase give rise to a textured and periodically modulated surface potential. A 1D lateral hBN (111)/(337) heterostructure arises, featuring defect‐free boundaries and significant band offsets. Since size tunability of phases can be gained by selecting the Rh vicinal plane, we envision the hBN (111)/(337) faceted system as a model platform to mold and probe 1D phonon–polariton excitations in the THz regime,^[^ ^15^ ^]^ to achieve selective growth of atoms, aggregates, and molecular adsorbates for organic opto‐electronics and catalysis,^[^ ^5^, ^16^ ^]^ and to tailor the 3D stacking with other 2D materials, such as graphene.^[^ ^7^ ^]^

## 2 hBN‐Induced Faceting across a Rh Curved Surface

**Figure 2:**
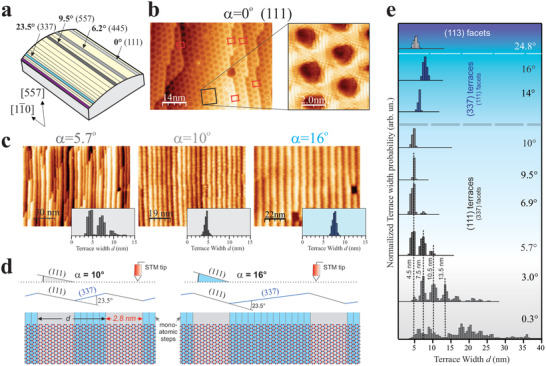
Evolution of the hBN nanostriped system on a curved Rh surface. a) Sketch of the c‐Rh sample with some vicinal *α* angles and corresponding crystal planes indicated. b) 4K‐STM image and zoom‐in near the hBN‐covered Rh(111) plane (*α* = 0). c) 300K‐STM topographies and terrace width histograms at characteristic *α* angles: (111) terraces reveal discretized hBN nanomesh quanta (*α* = 5.7°), a single nanomesh quantum (*α* = 10°), and hBN‐(337) nanostripes (*α* = 16.0°). d) Description of the STM scan over the hBN/Rh interface at *α* = 10° and *α* = 16°, where hBN‐(111) and hBN‐(337) nanostripes are respectively imaged as “terraces.” e) Terrace width distribution histograms across the curved hBN/c‐Rh surface. At low *α* (gray region), peaks correspond to multiples of the (111) nanomesh, down to a single quantum at *α* = 10°. Above *α* = 10° (blue region), the distribution shifts to the right side, reflecting the increasing width of the (337) facets. Beyond the (337) plane (*α* > 23.5°, purple region), (113) faceting arises.

We examined striped hBN monolayers over an ample range of Rh vicinal orientations with a curved Rh surface (c‐Rh). The sample is schematically depicted in **Figure**  **2**a. It is a cylindrical section of a Rh single crystal with the cylinder axis along $\left[1\bar{1}0\right]$ direction. The curved surface spans all vicinal surfaces (*α* angle) from the high symmetry (111) (*α* = 0) at the right edge of the sample, passing through the (557) plane (*α* = 9.5°) at the sample center, and reaching the (337) surface (*α* = 23.5°) near the left side edge. The freshly prepared surface is heated to *T* = 1000K, and then exposed to borazine, leading to full monolayer coverage of hBN across the entire curve, as judged from scanning tunneling microscopy (STM) and low energy electron diffraction (LEED). At the Rh(111) plane, this results in a hBN nanomesh structure,^[^ ^19^ ^]^ which arises due to atomic lattice mismatch and variable bonding between the hBN layer and the Rh substrate.^[^ ^20^ ^]^ Images in Figure  2b, acquired with a 4K‐STM, show the atomically resolved structure close to the Rh(111) plane and near substrate steps. We observe that (111) terraces are delimited by steps corresponding to Rh(111) single step height, with upper and lower terraces coated with a continuous hBN film. Within (111) terraces, the lattice constant of the hexagonal nanomesh is *l* = 3.2 nm, agreeing with former studies,^[^ ^19^, ^20^ ^]^ while across the steps the pore lattice is coherently connected (red rectangles), indicating that hBN forms a continuous layer that carpets atomic steps (see also Supporting Information for a graphical demonstration).

Away from the (111) direction, the hBN monolayer interacts with the step array of the substrate elastically, leading to faceting. We examined such interaction across the entire hBN‐covered c‐Rh surface by STM at *T* = 300 K. Figure 2c shows STM images acquired at three characteristic points at *α* = 5.7°, 10°, and 16°, together with their respective terrace‐width *d* distribution histograms. At *α* = 5.7° we observe straight and parallel hBN nanostripes, which fit in Rh(111) terraces of discrete sizes, separated by bunches of one to four Rh atomic steps (see Supporting Information). The histogram points to an interface that is broken up in a random distribution of hBN‐covered (111) terraces of width *d* ≈ 4.6, 7.1, and 9.6 nm, alternated with step bunches. Consequently, the terrace width increases approximately in multiples of the nanomesh periodicity in the perpendicular direction to the steps (nanomesh quantum Δ*d* ≈ *l* × cos 30° = 2.8 nm). The lowest value *d* ≈ 4.6 nm agrees with the nanomesh quantum plus the effective width of the step bunch (1.8 nm). The central panel of Figure  2c corresponds to the surface at *α* = 10°. At this vicinal angle, the hBN monolayer becomes a periodic structure with *d* ≈ 5 nm of nanostripes delimited by bunches of 3–5 monatomic steps and, most important, with minimal size fluctuations around one nanomesh quantum.

The right‐hand side STM micrograph of Figure  2c displays the growth of hBN on a densely stepped c‐Rh area at *α* = 16°. In the STM image we observe well‐defined hBN nanostripes, but compared with the *α* = 10° image, terraces appear wider with *d* ≈ 8 nm and the step direction has reversed from uphill to downhill. This effect is schematically explained in Figure  2d. At *α* = 10°, (111)‐oriented nanostripes are wider than hBN‐covered step bunches, and hence (111) nanostripes appear as terraces in the STM image. At *α* = 16° (111) nanostripes are narrower than stepped facets, which are thus imaged as terraces, while hBN‐(111) nanostripes appear as downhill steps. 4K‐STM, ARPES, and LEED experiments (see below) prove that the orientation of the stepped facets is the Rh(337) direction. This transition in the orientation of the apparent terrace beyond *α* = 10° is reflected in the terrace‐width distribution analysis performed across the entire surface, shown in Figure  2e. This provides an unified picture of the hBN/c‐Rh interface. In the *α* < 10° range, (111)‐oriented terraces are discrete multiples of the perpendicular nanomesh. With increasing *α*, the center of gravity of the terrace width distribution shifts toward the first nanomesh quantum. For *α* > 10°, hBN‐(337) nanostripes turn into apparent terraces, and hence, the histogram shifts to the right side reflecting the increasing (337) terrace width. Importantly, a sharp size distribution remains, demonstrating that hBN‐(337) nanostripes have a single characteristic width at each vicinal angle over the 10° < *α* < 23.5° range. Beyond 23.5°, hBN‐(111) nanostripes disappear and hBN‐covered (113) facets emerge.

## 3 The hBN‐Covered Rh(111)/Rh(337) Interface

**Figure 3:**
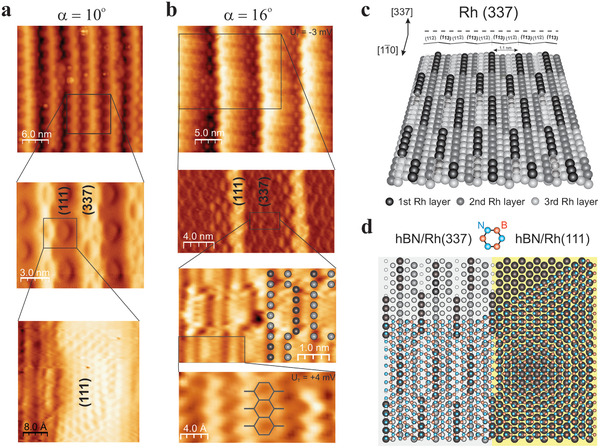
Atomic structure of hBN‐covered Rh(111)‐(337) facets. a) and b) 4K‐STM topographies (top) and progressive filtered zoom‐ins (second to bottom) at *α* = 10° and *α* = 16°, respectively. b) The atomic positions of the step in the reconstructed (337) surface appear overlaid (third image), as well as the hBN lattice (fourth image). c) Model for the Rh(337) surface with selective step atom removal (white dashed balls). Black balls mark the outermost surface atoms, while gray balls become lighter in successive surface levels. d) Model for a continuous hBN monolayer (blue‐red balls and sticks) in a coherent (111)/(337) lateral interface. In hBN/Rh(111), the coincidence lattice leads to nanomesh pores around N/Rh on‐top sites. In hBN/Rh(337), black balls mark the out‐protruding step atoms, which correspond to N/Rh on‐top sites of the hBN overlayer.

Next we focus on the hBN‐induced Rh(111)‐Rh(337) periodic faceting, which exhibits straight, and sharply defined hBN nanostripes with coherent coupling between (111) and (337) phases. The atomic arrangement at the hBN/Rh interface of each phase is investigated in detail using 4K‐STM. Images in **Figure**  **3**a,b have been acquired again at *α* = 10° and *α* = 16° on the c‐Rh surface. The larger view in top panels corresponds to the direct topography, while lower panels are image derivatives showing progressive zoom‐ins acquired at varied tip bias. Such image processing removes the hill‐and‐valley modulation, allowing us to define the lateral (111)/(337) interface, which shows no sign of discontinuity. From 10° to 16°, (337) terraces widen from *d*_337_ = 2 nm to ≈8 nm, while the (111) nanostripe width barely changes. The internal structure of each phase is visualized in the bottom panels of Figure  3a,b. This includes the hBN atomic lattice in both hBN‐(111) and hBN‐(337) nanostripes, as indicated with the overlaid structure. The hBN‐(111) nanostripe shows a wavy wire running parallel to the step [bright protrusion in top panel of (b)], with half‐pores at nanostripe boundaries. The hBN‐(337) nanostripe exhibits a characteristic texture, with the *l* = 3.2 nm nanomesh periodicity in the parallel direction, and a reduced one of 1.1 nm perpendicular to the steps, both defining a 2D structure with uniaxial symmetry. Such nanoscale texture in the hBN layer is explained by the periodic re‐arrangement of the Rh(337) substrate steps described in Figure  3c.

Adsorption‐induced faceting of vicinal surfaces is driven by the optimal registry of the adlayer atomic lattice with the step spacing, such as to reduce interface strain.^[^ ^21^ ^]^ For hBN/metal systems, characteristic facets are expected for step periodicities that match an entire number of hBN cells.^[^ ^22^ ^]^ In the hBN/Rh system, we may compare the step spacing in the Rh(337) plane with the distance between contiguous N rows in hBN. As shown in Figure  3c, the (337) plane can be viewed as an alternating sequence of (112) and (113) terraces, resulting in a *l*_337_ = 1.1 nm lattice constant perpendicular to the steps. This closely corresponds to five rows of N atoms (5 × *a*_hBN_ × cos 30° = 1.08 nm) in the hBN monolayer with *a*_hBN_ = 2.504Å being the hBN lattice constant. Thus, by slightly (2%) stretching the hBN monolayer, only N atom rows are in coincidence with the out‐protruding step‐edge. Yet, the mismatch along the steps leads to periodic off‐registry (strained) N‐bridge positions. The substrate reconstruction proposed in Figure  3c solves this problem.

The Rh(337) unit cell contains two rows of step atoms at different levels, with the outermost one corresponding to the downhill step of the (112) terrace. As shown in Figure  3c, removal of 7 out of 12 step atoms in this row (dashed‐white transparent balls) reverses the (112)–(113) series and moves the contiguous step segments at the topmost surface position. Now, the outermost surface layer (black balls) is made of 5–6 step‐atom segments, defining a 2D lattice identical to that imaged in Figure  3b. In Figure  3d we overlay the hBN lattice on top of a (111)/(337) interface. The Rh(337) step re‐arrangement has the double effect of eliminating 7 off‐registry N‐bridge step sites, while creating six new registry N on‐top step sites. Thus, the Rh(337) reconstruction relieves strain by improving registry, making the hBN/Rh interface more robust through exclusive N/Rh on‐top bonding. Note that the *l* = 3.2 nm nanomesh periodicity remains along the steps, enabling the coherent coupling with neighboring hBN‐(111) nanostripes, which in turn explains the smooth coating of the faceted substrate (see also Figure S8, Supporting Information for a graphical explanation). Similar hBN/Rh step‐lattice registry and density of N on‐top step anchoring points can be obtained when considering separate hBN/Rh(112) and hBN/Rh(113) interfaces, as shown in Figure S3, Supporting Information. However, in (112) and (113) planes, off‐registry N bridge‐bonding to step atoms remains, in contrast to the sole N on‐top bonding of Figure  3c, which only the Rh(337) plane allows.

## 4 Uniaxial *π* Band Symmetry in the hBN‐(337) Surface

**Figure 4:**
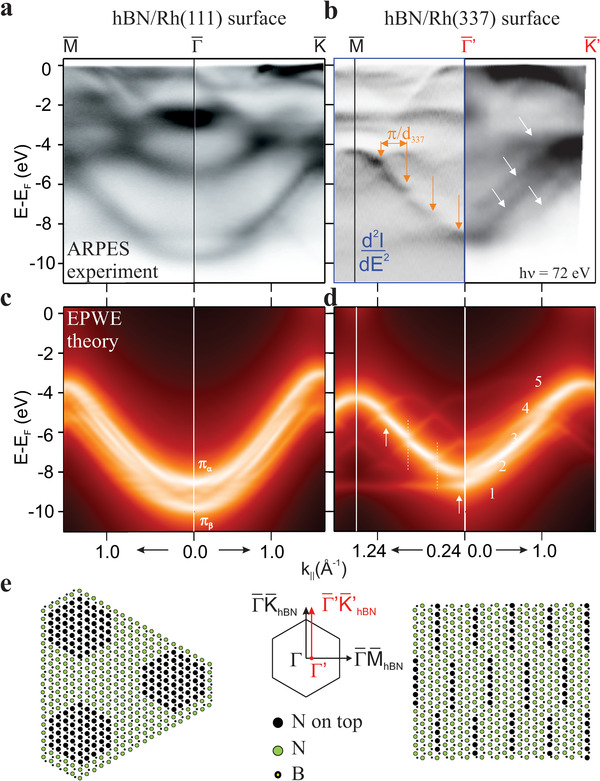
hBN electron bands on Rh(111) and Rh(337). a,b) Experimental hBN bands along the $\bar{\Gamma }\bar{M}$, $\bar{\Gamma }\bar{K}$, and ${\bar{\Gamma }}^{\prime }{\bar{K}}^{\prime }$ directions measured with ARPES in the Brillouin zones of hBN/Rh(111) and hBN/Rh(337) as indicated in (e). For hBN/Rh(337) in (b), arrows mark mini‐gaps at *n* × *π*/*d*_337_ Brillouin zone edges along $\bar{\Gamma }\bar{M}$ and the *π* band manifold along ${\bar{\Gamma }}^{\prime }{\bar{K}}^{\prime }$. c,d) Theoretical hBN bands calculated with the EPWE model, assuming the different atomic potentials at pores/steps (N black) and wires (N green) shown in (e). The fivefold *π* band splitting along ${\bar{\Gamma }}^{\prime }{\bar{K}}^{\prime }$ is numbered from bottom to top in (d).

The structurally different hBN/Rh(111) and hBN/Rh(337) phases exhibit distinct electronic bands, which we display in **Figures** **4**a,b. The (111)‐nanomesh modulation triggers the splitting of all hBN electron levels,^[^ ^19^, ^23^ ^]^ from vacuum to valence and core bands, in contrast to the simple sequence of electronic states in non‐interacting or registry hBN monolayers.^[^ ^24^ ^]^ Figure  4a shows the electron bands measured with ARPES along the $\bar{\Gamma }{\bar{M}}_{\mathrm{hBN}}$ and $\bar{\Gamma }{\bar{K}}_{\mathrm{hBN}}$ directions in the hBN/Rh(111) system (*α* = 0). The Rh substrate emission dominates the spectrum from the Fermi energy down to −3 eV, while *σ* and *π* bands of hBN appear below this energy. The characteristic *π*_*α*_–*π*_*β*_ splitting is observed,^[^ ^23^ ^]^ due to the different interaction with the Rh substrate at pores and wires.^[^ ^25^ ^]^ As explained from density‐functional‐theory (DFT),^[^ ^23^, ^25^ ^]^ the highly bound *π*_*β*_ band originates in the highly interacting on‐top N atoms in the pores, while the *π*_*α*_ branch comes from off‐registry N atoms in the wires.

The pore/wire nature of the *π*_*α*_/*π*_*β*_ split‐bands in the hBN/Rh(111) interface is well captured in our electron‐plane‐wave‐expansion (EPWE) and photoemission model.^[^ ^26^ ^]^ Using only four parameters, namely, the different scattering potentials at N and B atoms, a rigid potential shift Δ*V* from wires to pores (first Fourier component of the nanoscale potential), and an effective mass m*, we fit the experimentally observed energies at $\bar{\Gamma }$, $\bar{M}$, and $\bar{K}$ points, and obtain the theoretical bands of Figure  4c (see also Figure S4, Supporting Information for more details). We indeed find that a *π* band splitting arises for Δ*V* < 0. To fit ARPES bands we require Δ*V* = −1.95 eV and m* = 1.03· *m*_*e*_, with *m*_*e*_ being the free electron mass (see **Table** **1**). Actually, due to the (13 × 13) periodicity, the simple EPWE model delivers multiple *π* band replicas within the hBN Brillouin zone, which are averaged out by introducing broadening in the photoemission probability. Note that experimental bands in Figure  4a only mirror the hexagonal symmetry of the atomic lattice, but do not show nanomesh umklapps, sometimes seen for graphene/metal moiré systems.^[^ ^27^, ^28^ ^]^

**Table 1:** *π* band properties in hBN/metal systems. *π* band bottom (${\bar{\Gamma }}_{\min}$), top (${\bar{K}}_{\max}$), and bandwidth (Δ_Γ*M*_) measured with ARPES in hBN/metal systems. Data for hBN/Ni(111) and hBN/Au/Ni(111) are from Ref. [24]. 〈*V*_N_〉_*w*, *p*_ and m* respectively are the average surface potential at N atoms for wires and pores, and the effective mass used in the EPWE model, which renders the semiconducting gap (*E*_F_ gap). All magnitudes are expressed in eV

Interface	${\bar{\Gamma }}_{\min}$	${\bar{K}}_{\max}$	Δ_Γ*M*_	*E*_*F*_ gap	<*V*_N_>_*w*_	<*V*_N_>_*p*_	<m*>
hBN/Au/Ni(111)	−8.2	−2.22	4.87	5.08	0.0	0.0	1.00
hBN/Ni(111)	−10.0	−4.45	4.53	5.08	−1.690	−1.690	1.05
hBN/Rh(111)	−9.8 ±0.2	−3.0 ±0.2	4.4 ±0.1	4.20	−0.325	−2.275	1.03
hBN/Rh(337)	−8.8 ±0.2	−3.3 ±0.2	2.86 ±0.1	4.87	0.175	−1.775	1.10

In the hBN/Rh(337) bands, the uniaxial symmetry brought in by the nanoscale texture is reflected in the band structure, shown in Figure  4b. Data correspond to *α* = 24°, that is, very close to the Rh(337) surface. Due to experimental restrictions we were not able to measure in the exact $\bar{\Gamma }{\bar{K}}_{\mathrm{hBN}}$ direction but only close to it as indicated in the middle panel of Figure  4e. Nevertheless, we now observe a striking asymmetry from the perpendicular ($\bar{\Gamma }{\bar{M}}_{\mathrm{hBN}}$) to the parallel (${\bar{\Gamma }}^{\prime }{\bar{K}}_{\mathrm{hBN}}^{\prime }$) direction along substrate steps. Perpendicular to the steps, the main dispersing feature is broken up in segments, separated by ≈0.3 meV mini‐gaps (see arrows; the second derivative of the image enhances gap visualization, see Supporting Information), whereas in the parallel direction the *π* band splits in a set of continuous sub‐bands (inclined arrows). Such distinct band topology is correctly reproduced in EPWE calculations of Figure  4d, which are based on the atomic model depicted in Figure  4e. Note that despite the apparent atomic complexity, the hBN/Rh(337) interface is simple in terms of bonding, since all the outermost Rh step atoms bind to N in atop positions. For the EPWE model, it is thus reasonable to assume the same surface potential reduction at N atoms on steps that was used for pores, Δ*V* = −1.95 eV. In excellent correspondence with the experimental observations in Figure  4b, the model reveals the presence of a fivefold *π* band splitting at $\bar{\Gamma }$, which leads to a set of dispersing bands along $\bar{\Gamma }{\bar{K}}_{\mathrm{hBN}}$, and to five mini‐bands separated by mini‐gaps at Rh(337) Brillouin zone edges (*n* × *π*/*d*_337_) along $\bar{\Gamma }{\bar{M}}_{\mathrm{hBN}}$.

The hBN/Rh(337) system represents the first successful case of uniaxial symmetry imprinted onto the electronic structure of the hBN monolayer. In pursue of this objective, (110)‐oriented metal surfaces were tested in the past,^[^ ^29^, ^30^, ^31^, ^32^ ^]^ but the hBN film was observed to break up in rotational phases. On Rh(110),^[^ ^33^ ^]^ hBN nanostriped domains appear, reflecting the scarcity of favorable interface (N on‐top) bonding sites compared to reconstructed Rh(337). Nevertheless, away from high symmetry directions, other hBN/metal surface combinations should exist with optimal lattice matching. Uniaxial vicinal planes with different terrace orientations, such as (110), (110), or (201), as well as other metals, such as Pt, Pd, Ru, or Cu should be explored.^[^ ^5^ ^]^ hBN‐induced faceting appears to be the rule rather than the exception,^[^ ^22^ ^]^ and this can serve to pinpoint new “magic,” well‐matched surfaces, such as Rh(337), and to find nanostriped patterns with varied textures.

In Table 1 we summarize the *π* band properties for fundamental hBN/Rh systems, namely, free‐standing (Au/Ni), 2D atomic‐registry [Ni(111)], 2D nanomesh [Rh(111)], and 1D step‐registry [Rh(337)], from which we derive some trends. The *π* band minimum at $\bar{\Gamma }$ (${\bar{\Gamma }}_{\min}$) is connected to the ratio of substrate‐interacting N atoms in top positions to the total number of N atoms. Such ratio increases from 20% in Rh(337) steps, to 40% in Rh(111) pores, and to 100% in lattice‐matched Ni(111), and directly correlates with the average 〈*V*_N_〉 potential in the EPWE model. Despite the strong difference in interface bonding, the hBN gap remains unchanged in Ni and Au/Ni, whereas nanoscale texturing in Rh(111) and Rh(337) effectively reduces the gap.

## 5 Electronic States in Lateral hBN (111)/(337) Heterostructures

**Figure 5:**
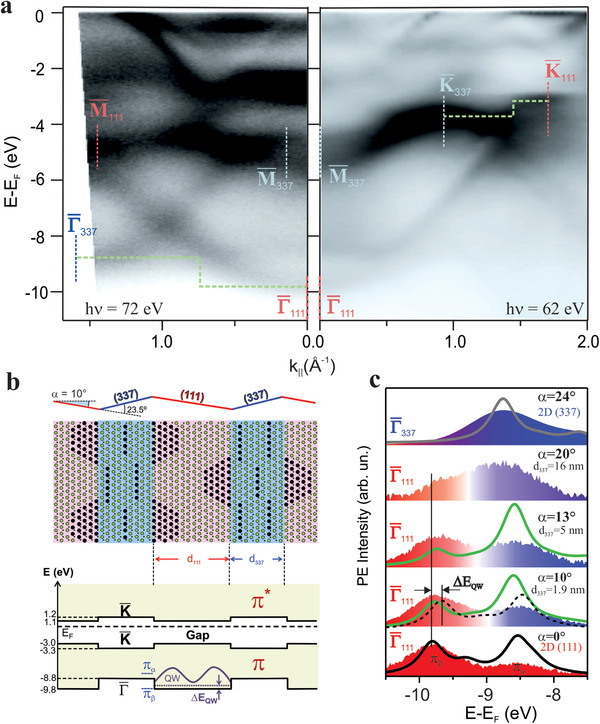
Electronic states in lateral hBN heterostructures. a) Electron bands for the hBN (111)/(337) superlattice measured with ARPES (h*ν* = 72 eV) at *α* = 10° in the hBN/c‐Rh sample. The faceted geometry leads to separate emission from (111) and (337) facets. The respective $\bar{\Gamma }$, $\bar{M}$, $\bar{K}$ symmetry points and the resulting heterostructure offsets are indicated. b) Atomic model and band diagram for the hBN (111)/(337) lateral heterostructure, as determined from data and calculations of Figure  4. c) Evolution of the ARPES intensity at ${\bar{\Gamma }}_{111}$ in the (111)/337 nanostriped system at increasing *α*. The spectrum on top corresponds to the ${\bar{\Gamma }}_{337}$ point of the 2D hBN‐(337) system. Lines are EPWE spectra at $\bar{\Gamma }$ for heterostructures of fixed *d*_111_ = 2.8 nm and variable *d*_337_. ΔE_QW_ marks the expected EPWE shift of *π*_*β*_ in fully decoupled 2.8 nm *d*_111_ nanostripes.

The straight boundaries with coherent atomic bonding, together with the distinct band properties of (111)‐ and (337)‐oriented hBN nanostripes define an ideal atom‐thick, lateral semiconductor heterostructure. In **Figure**  **5**a we show electron bands as obtained from ARPES measurements at *α* = 10° along $\bar{\Gamma }{\bar{M}}_{\mathrm{hBN}}$ and $\bar{\Gamma }{\bar{K}}_{\mathrm{hBN}}$, where the superlattice is formed by *d*_111_ = 2.8 nm and *d*_337_ ≈2 nm hBN nanostripes. This nanostriped system is depicted in Figure  5b, together with its band diagram, where we have assumed the energies of Table 1 to define valence and conduction band offsets. Interestingly, the hill‐and‐valley geometry of the surface plane leads to separate intensity from (111)‐ and (337)‐oriented facets in ARPES scans (see Figure S6, Supporting Information for details). This allows us to track the evolution of the *π* band emission from each phase, and hence to prove (111)/(337) electronic coupling. This is done for the 2.8‐nm‐wide *d*_111_ nanostripe in Figure  5c. Red‐blue shaded spectra correspond to *k*_||_ = 0 in the (111) plane (${\bar{\Gamma }}_{111}$ point), whereas green curves are EPWE calculations for the heterostructure of Figure  5b, assuming *d*_337_ nanostripes of increasing width. The spectral density clearly evolves from the hBN‐(111) system (bottom) to hBN‐(337) (top) as a function of *α*, that is, from the 2D (111) nanomesh to increasingly sparse *d*_111_ = 2.8 nm nanostripes. Because of the 1 eV valence band offset at the interface, the *π*_*β*_ peak, which lies below the band minimum in the (337) plane (Γ_337_), is weakly coupled to the (337) bands. Consequently, *π*_*β*_ behaves as a (111)‐terrace‐like state, which shifts up in energy and has its intensity reduced when the (111) surface evolves into a narrow stripe. In contrast, the *π*_*α*_ feature that lies above the Γ_337_ minimum resonates with the (337) band and hence remains intense. Note that we are exclusively probing emission from *d*_111_‐oriented areas of the surface. Thus, Figure  5c demonstrates that *d*_111_ nanostripes embedded within large *d*_337_ areas at *α* = 20° produces emission that is coherent with the hBN‐(337) system. In fact, the asymmetric intensity variation of *π*_*α*_ and *π*_*β*_, as well as the small (50 meV) shift of *π*_*β*_, are reproduced in our calculations for electronically‐coupled 111/337 interfaces. In contrast, our calculations permit us to discard a fully decoupled, quantum‐well‐like *d*_111_ nanostripe (black dotted spectrum in Figure  5c), which would produce spectra exhibiting a symmetric *π*_*α*_–*π*_*β*_ intensity quenching, and a substantially larger peak shift (Δ*E*_QW_ = 150 meV).

## 6 Conclusions

We have explored a novel approach to nanopatterning a material with practical technological impact, namely growing hBN monolayers on vicinal surfaces. Growth of hBN on vicinal Rh(111) leads to periodic (111)/(337) faceting, triggered by excellent atomic registry of hBN with a step‐reconstructed (337) surface plane. The hBN film forms a continuous layer over the faceted system with alternated (111) and (337) phases, each exhibiting a characteristic internal texturing, and strain‐free atomic bonding at the mutual interface, thereby defining an electronically‐coherent hBN lateral heterostructure.

In the context of the rich phenomenology exhibited in hBN/metal interfaces,^[^ ^5^ ^]^ we devise a vast number of possibilities for vicinal surfaces. These are known to drive the nucleation and growth of azimuthally‐oriented hBN flakes,^[^ ^34^, ^35^ ^]^ and here we show that they also trigger 1D texturing, as depicted in Figure 1. Beyond the (111)/(337) system, other hBN/metal orientations may also exhibit faceting with distinct electronic properties and textures, and this could be systematically explored with curved surfaces. The nanoscale corrugation of the surface potential can help to imprint, for example, a periodic modulation of energy and filling of molecular levels in organic adsorbates.^[^ ^16^ ^]^ It can also have a strong influence in epitaxial growth, for example, in the vertical stacking of transition metal dichalcogenides (TMDs) or graphene, where it may help to tune, in general, electronic properties^[^ ^36^ ^]^ and, in particular, the twist angle.^[^ ^7^ ^]^ The potential for optoelectronics of such 2D material stacking should also be explored for applications, such as band structure modulation of graphene and TMDs. The hBN (111)/(337) heterostructure is also an ideal platform to manipulate phonon‐polariton excitations and their dynamics.^[^ ^15^ ^]^ The band structure modulation is expected to affect the optical response of the material, thus adding an appealing alternative to the suite of existing possibilities in the THz region.

## Conflict of Interest

The authors declare no conflict of interest.

## Supporting information

Supporting Information

## Data Availability

Research data are not shared.
