# Supplementary material for: Atomically‐Precise Texturing of Hexagonal Boron Nitride Nanostripes
Source: Adv Sci (Weinh). 2021 Jul 22;8(17):2101455. doi: 10.1002/advs.202101455 (PMC8425884; doi:10.1002/advs.202101455)
Supplement: Supplementary file 1 — Supporting Information [file ADVS-8-2101455-s001.pdf]

© 2021 Wiley-VCH GmbH

ADVANCED SCIENCE
Open Access

Supporting Information

for *Adv. Sci.*, DOI: 10.1002/advs.202101455

Atomically-precise texturing of hexagonal boron nitride nanostripes

Khadiza Ali,[†] Laura Fernández,[‡] Mohammad A. Kherelden,[¶] Anna A. Makarova,[§] Igor Píš,[‖,⊥] Federica Bondino,[‖] James Lawrence,[#] Dimas G. de Oteyza,[#] Dmitry Yu. Usachov,[@] Denis V. Vyalikh,[#] F. Javier García de Abajo,[∇,††] Zakaria M. Abd El-Fattah,[¶] J.Enrique Ortega,[‡,†,#] and Frederik Schiller[*,†,#]

Atomically-precise texturing of hexagonal boron nitride nanostripes

Khadiza Ali,[†] Laura Fernández,[‡] Mohammad A. Kherelden,[¶] Anna A. Makarova,[§] Igor Píš,[‖,⊥] Federica Bondino,[‖] James Lawrence,[#] Dimas G. de Oteyza,[#] Dmitry Yu. Usachov,[@] Denis V. Vyalikh,[#] F. Javier García de Abajo,[∇,††] Zakaria M. Abd El-Fattah,[¶] J.Enrique Ortega,[‡,†,#] and Frederik Schiller[*,†,#]

† *Centro de Física de Materiales CSIC/UPV-EHU-Materials Physics Center, E-20018 San Sebastián, Spain*

‡ *Universidad del País Vasco, Dpto. Física Aplicada I, E-20018 San Sebastián, Spain*

¶*Physics Dept., Faculty of Science, Al-Azhar University, Nasr City, E-11884, Cairo, Egypt*

§ *Institut für Festkörper- und Materialphysik, TU Dresden, D-01062 Dresden, Germany*

‖ *IOM-CNR, Laboratorio TASC, I-34149 Trieste, Italy*

⊥ *Elettra - Sincrotrone Trieste S.C.p.A., I-34149 Trieste, Italy*

Donostia International Physics Center, E-20018 San Sebastián, Spain

@*St. Petersburg State University, 199034 St. Petersburg, Russia*

△*Ikerbasque, Basque Foundation for Science, 48013 Bilbao, Basque Country, Spain*

∇ *ICFO-Institut de Ciencies Fotoniques, The Barcelona Institute of Science and Technology, 08860 Castelldefels (Barcelona), Spain*

†† *ICREA-Institució Catalana de Recerca i Estudis Avançats, Passeig Lluís Companys 23, 08010 Barcelona, Spain*

E-mail: frederikmichael.schiller@ehu.es

Supporting Information Available

The supplementary information file contains:

Materials and Methods

Supplementary Figures S1-S8

Supplementary information and data are provided to further illustrate the STM analysis of hBN at the c-Rh(557) (Fig. S1), to show the evolution of the Low Energy Electron Diffraction (LEED) pattern (Fig. S2), to sketch the lattice matching of hBN to Rh(112), Rh(113), and Rh(337) planes (Fig. S3), to better explain the EPWE model illustrated with the hBN/Au/Ni(111) and hBN/Ni(111) cases (Fig.S4), to show the N 1s X-ray photoemission across the hBN-covered c-Rh(557) surface (Fig. S5), to show the full ARPES scan and explain the ARPES measurement geometry of the different facets (Fig. S6), to compare the direct intensity and the second derivative ARPES maps for the dispersion along $\bar{\Gamma}'\bar{M}$ in the hBN/Rh(557) system, and to graphically explain the moiré patterns arising upon carpeting both monatomic steps in the (111) surface, and the 111/557 heterostructure.

Materials and Methods

Experimental details

The Rh curved sample [c-Rh(557)] is sketched in Fig. 1(a) of the main text. Since the cylinder axis is parallel to the $[1\bar{1}0]$ direction, and the sample center is the (557) surface, vicinal surfaces with increasing density of close-packed steps with {100} (A-type) microfacets are found as one moves away from the (111) direction in one side of the sample to the other side of the sample, up to about 25.5° vicinal angle. The curved surface is prepared in ultra-high vacuum following the standard procedure applied to regular crystals, namely sputtering (Ar^+, 1 keV), annealing (1000 K), O_2-annealing (2×10^{-7} mbar O_2) cycles and short flashes to 1100 K. This procedure leads to a carbon-free surface exhibiting a sharp low energy electron

diffraction pattern across the whole sample.

Scanning tunneling microscopy (STM) experiments were performed at two different Omicron setups, one operating at $T = 300$K and another one at $T = 4$K. Angle-resolved Photoemission Spectroscopy (ARPES) experiments were carried out at a home set-up using HeIIα light (40.8 eV) and a Phoibos 150 SPECS analyzer (energy and angular resolution of 50 meV and 2°). Further, more detailed ARPES analysis was carried out as well as with a modified Scienta R3000 analyzer at the the CNR Beamline for Advanced diCHroism (BACH) at the Elettra synchrotron in Trieste (energy and angular resolution of 50 meV and 1°, multichannelplate detector acceptance angle ±10.5°). In the synchrotron experiment, also X-Ray Photoemission Spectroscopy (XPS) and X-ray absorption (XAS) total yield experiments were performed. All systems were equipped with Low Energy Electron Diffraction (LEED) that provided the way of checking the quality of the film and the local orientation at each sample position.

Statistical STM image analysis at 300 K

To image the curved surface at 300 K with the Omicron setup we used a tunneling current of ∼0.1 nA and a sample bias of ∼-1V or ∼+1.1 V. STM images were acquired at the center of surface areas exhibiting homogeneous step arrays in the μm scale. Terrace-width distribution probability $[P(d)]$ histograms in Fig. 2 of the main text and Fig. S1 correspond to a single STM frame, where sizes vary between 100×100 and 200×200 nm^2, which allows us to image between 15 and 40 steps within the the same frame at every α angle. Using a home-made IgorPro (http://www.wavemetrics.com) macro, we automatically analyzed every linescan (512 lines), eliminated outliers, and finally delivering 2000-10000 step-spacing events for a single histogram, which is found as a significant figure for a correct statistical analysis. The bar width in the histogram is 0.5 nm. For the $P(d)$ histograms (Fig. S1, top) there is no extra pre-processing. The mean terrace size $< d >$ is immediately deduced from the $P(d)$ histogram, and hence the local vicinal angle $\sin\alpha = h/ < d >$, where h is the step height in

each image. For the height distribution (Fig. S1, bottom), we used a local plane to flatten the surface.

Theoretical approach

The electron-plane-wave-expansion (EPWE) method was recently applied to graphene, carbon nanostructures, and organic molecules to successfully describe their π electronic structures within DFT level of accuracy.[1,2] Here, we extend this approach to pristine and nano-textured hBN systems. The potential landscape used for the calculations of pristine hBN systems is depicted in Fig. S4(a). The large (d_{B} = 1.59 Å) and small (d_{N} = 0.97 Å) circles define the boron and nitrogen atoms, respectively, each of them characterized by an inner potential $V_{\mathrm{in}} = 0$, while the yellow color stands for the interstitial region with $V_{\mathrm{out}} = 2.44$ eV. The conventional rectangular unit cell in Fig. S4(a), containing two sets of boron and nitrogen atoms, has lattice vectors of length $a = 2.5$ Å and $b = 4.33$ Å. We then solve the Schrödinger equation for this 2D potential landscape $V(x,y)$,

$$-\frac{\hbar^2}{2m^*}\nabla^2\psi(x,y) + V(x,y)\psi(x,y) = E\psi(x,y), \tag{1}$$

using the EPWE method detailed in Ref. 1. Here, $\psi(x,y)$ is the electron wave function and m^* denotes the electron effective mass. To achieve high convergence, we terminate the potential expansion at $g_{\mathrm{max}} \geq 15$ for the simple (1×1) structures, while for all nano-textured hBN structures g_{max} was set to 121, where g_{max} refers to the radius of a circle in which reciprocal space vectors are included in the calculation, in units of the inverse lattice constant.[1] For the freestanding hBN/Au/Ni(111) system, we require $m^* = 1.0\ m_{\mathrm{e}}$, and the Fermi energy ($E_F$) is chosen to set the $\bar{\Gamma}$-point energy at -8.2 eV.[3] For the interacting hBN/Ni(111) system, we use $m^* = 1.048$, m_e and ΔE_F = -1.69 eV to fit the relatively narrower $\bar{\Gamma}\bar{M}$ band width and the higher energy (-10.0 eV) $\bar{\Gamma}$-point, respectively. The resulting band structures (b) and simulated photoemission intensity (c) agree well with DFT calculations[4] and ARPES

measurements,[3] fairly reproducing the direct band gap (d) characteristic for a monolayer hBN.

The unit cells of the 2D *nanomesh* and (337) systems (Fig. 4 in the main text) are constructed by setting the potential difference between pores as well as on-step N atoms and the wire regions to ΔV = -1.95 eV, while the respective m^* and ΔE_F values that fit the experimental $\overline{\Gamma M}$ band width and the $\bar{\Gamma}$-point energy are given in Table 1 in the main text. For the (111)-(337) oriented stripes, the first Fourier component of m^* is chosen to describe such hybrid systems, while the remaining scattering parameters are kept to the same values of the pure *nanomesh* and (337) systems.

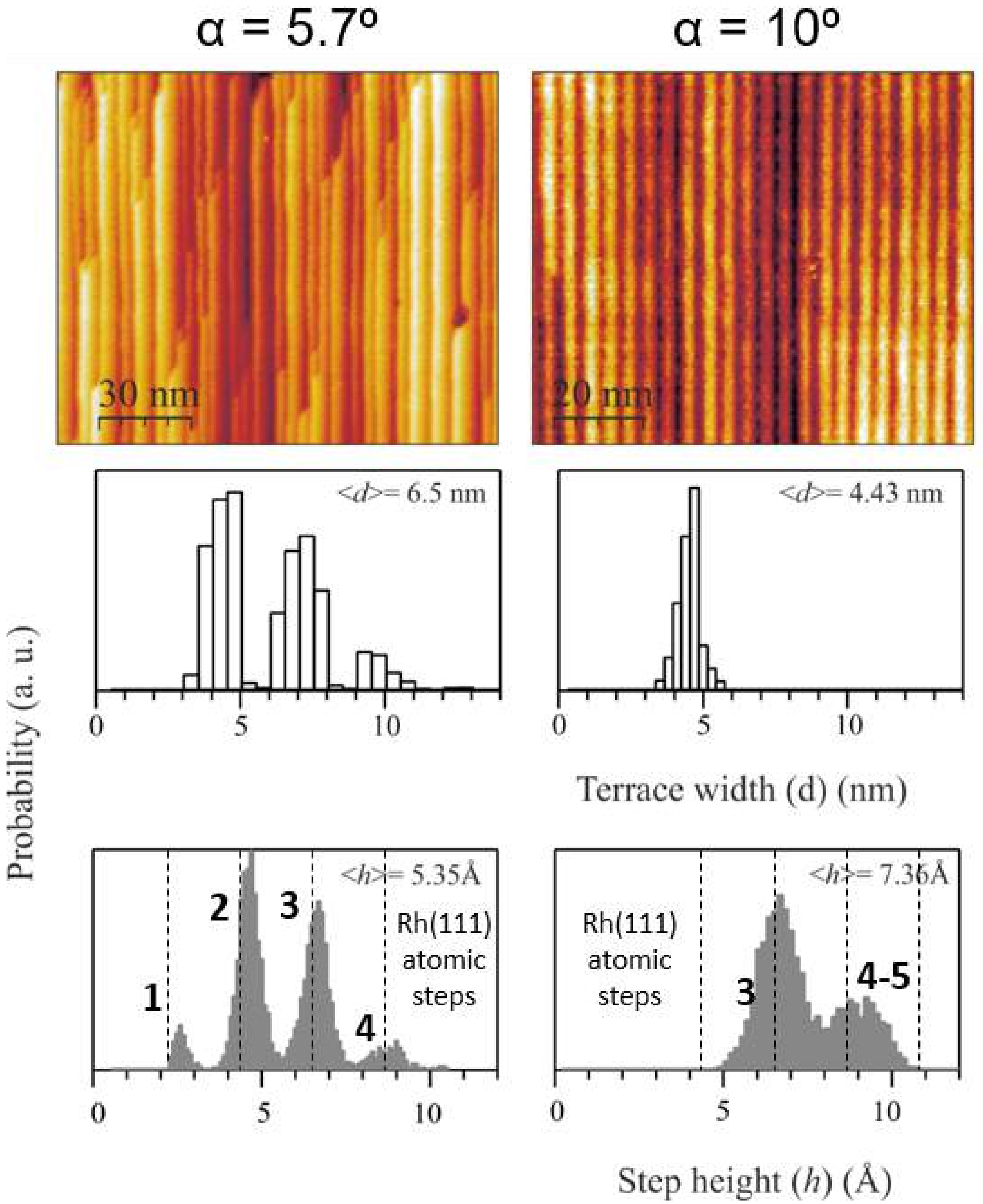

Figure S1: **Step height histograms.** STM images (top) and corresponding histogram analysis for terrace sizes (center) and step heights (bottom) performed at the hBN/c-Rh surface at $\alpha = 5.7°$ and $\alpha = 10°$. Discretization of the step size histograms reveals the formation of bunches of 1-4 Rh steps at $\alpha = 5.7°$ (Rh monatomic step is h=2.2 Å) and 3-5 steps at $\alpha = 10°$. The latter separate single quantum *nanomesh* stripes, with an effective periodic spacing of 4.4 nm (central panel), in agreement with Fig. 5 in the main text.

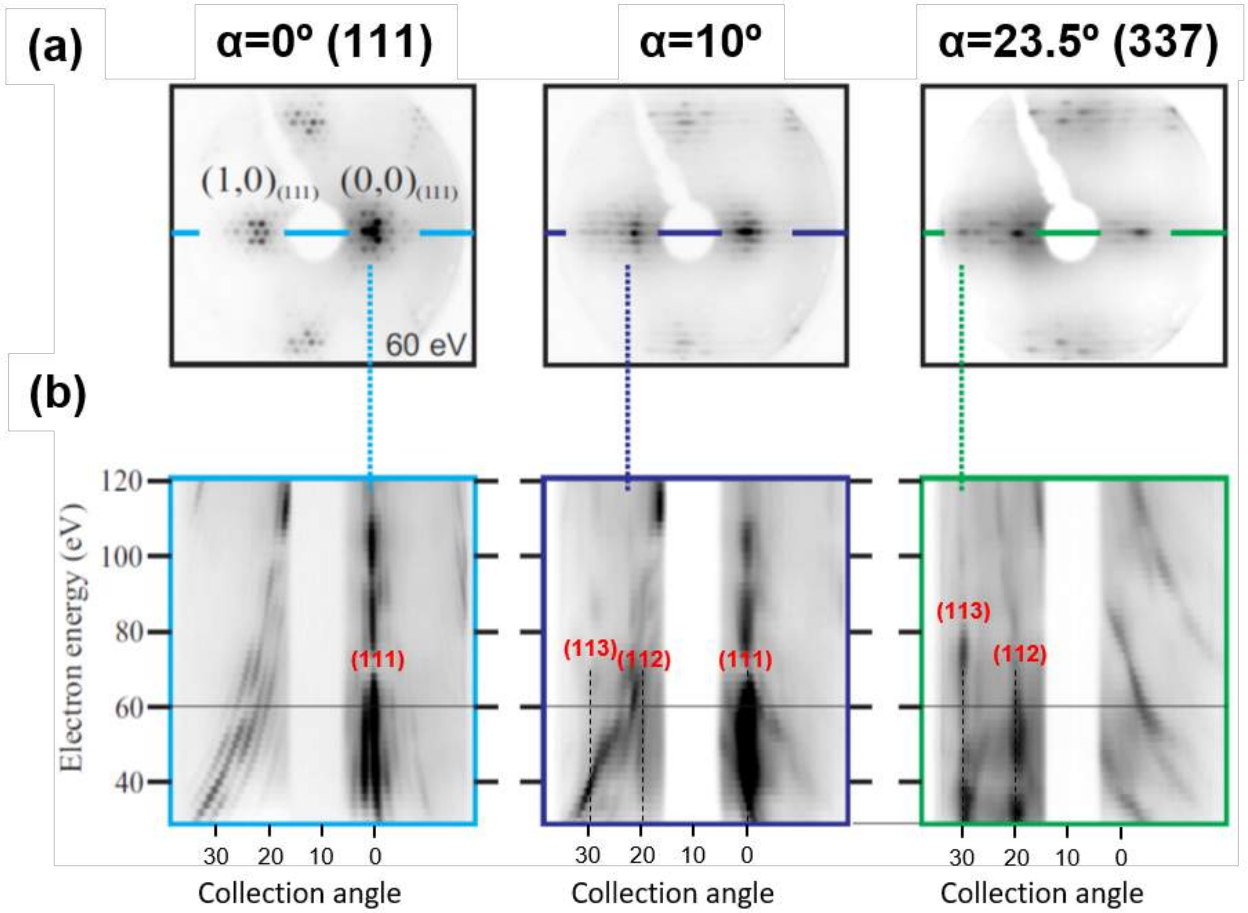

Figure S2: **Low Energy Electron Diffraction (LEED).** (a) LEED patterns at fixed primary electron beam energy E_p= 60 eV and (b) E_p-dependent intensity profiles across the (0,0) and (1,0) beams [marked as dashed lines in (a)] for the hBN/c-Rh system at three characteristic angles. The electron beam impinges with fixed crystal direction with respect to the bulk crystal. As the energy increases, (0,0) reflexions from distinct crystal planes appear as straight vertical lines. At $\alpha = 23.5°$ two separate lines arise at the (113) and the (112) crystal planes, which make up the (337) surface. At $\alpha = 10°$ the main reflexion belongs to the (111) terrace, while the emergent (113)/(112) pair agrees with the presence of (337) nanofacets.

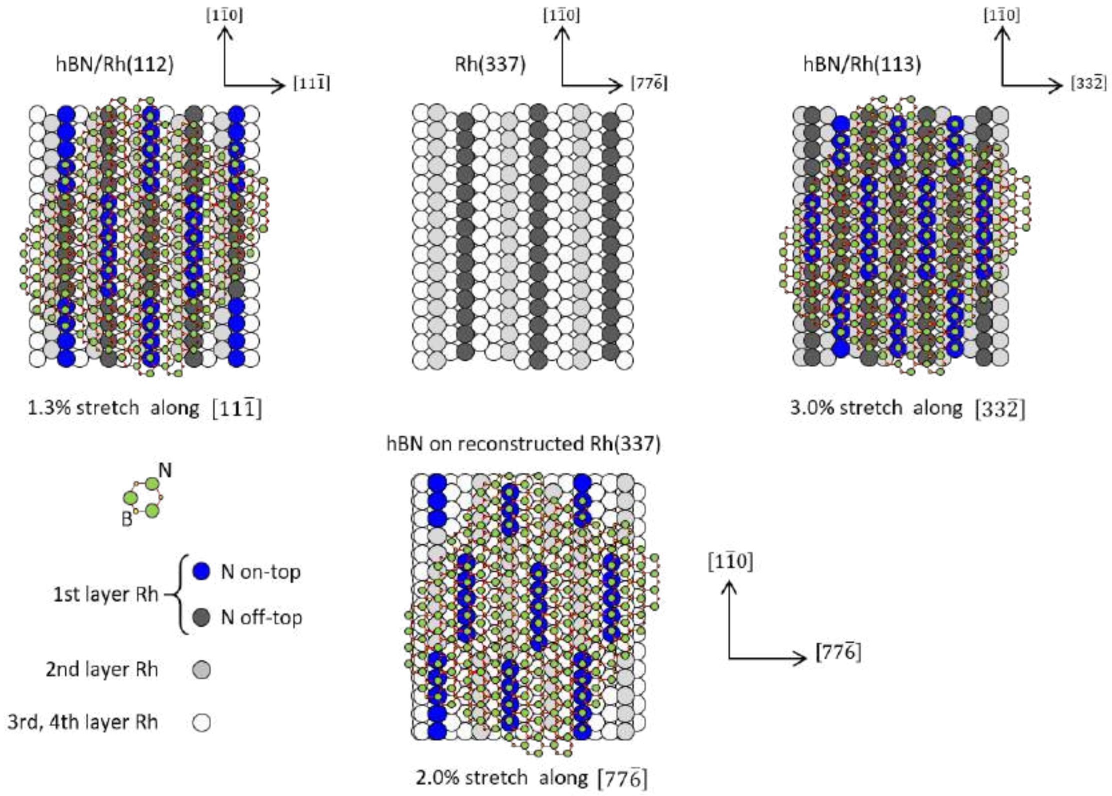

Figure S3: **Matching of hBN to Rh(112) and Rh(113) crystal planes.** Top view of Rh(112) (top left) and Rh(113) (top right) surfaces, covered with an hBN monolayer. Dark gray and blue circles represent the topmost Rh step atoms, with N atoms at bridge and on-top positions, respectively. To fit 3 and 2 N rows in the respective terrace, the hBN lattice has been stretched in the direction perpendicular to the steps by 1.3% and 3.1%, in order to achieve matching with the step lattice in each case. The central panel depicts pristine (top) and reconstructed (bottom) (337) surfaces. The latter has been covered with the hBN monolayer. Bridge adsorption at step edges is eliminated.

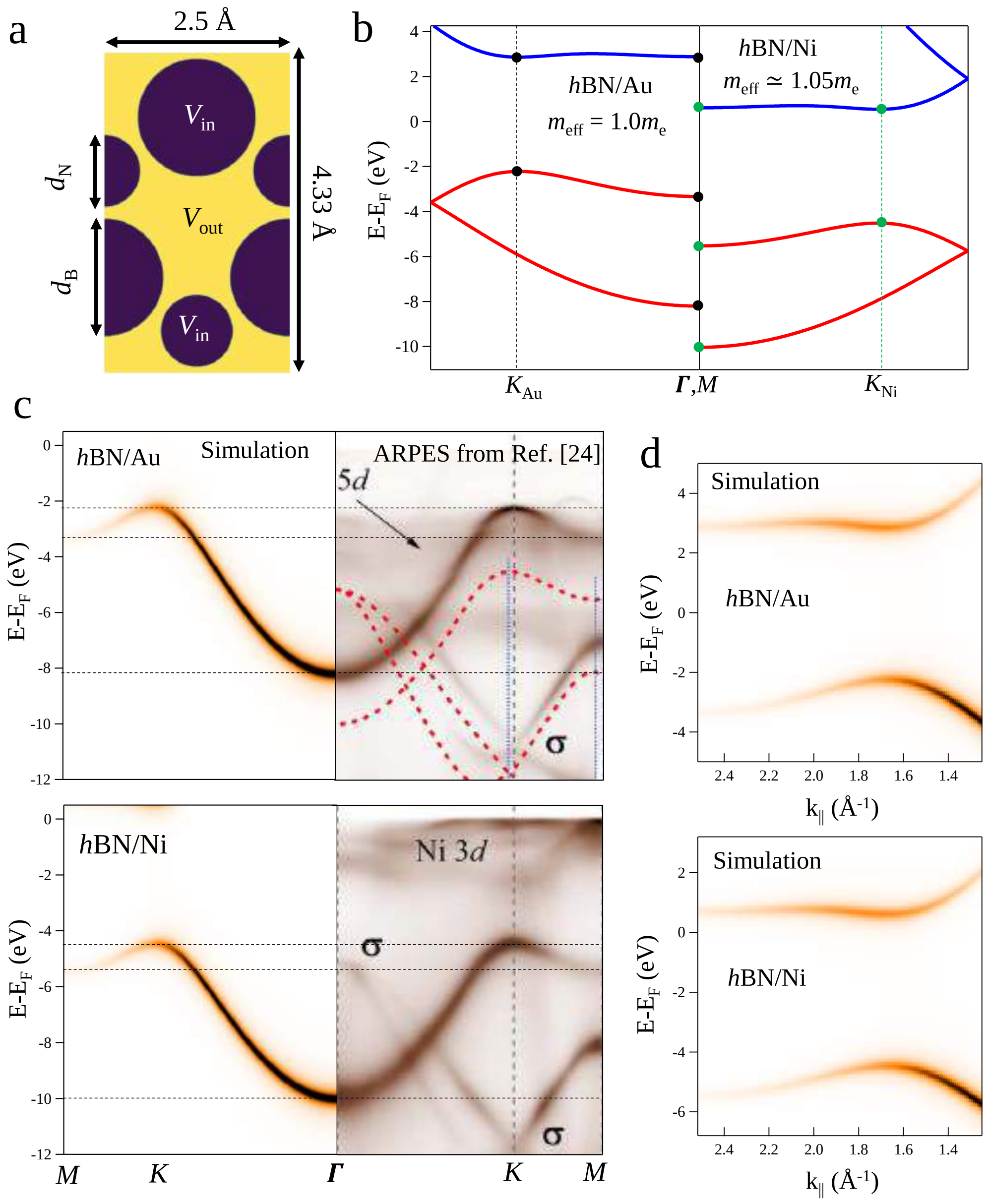

Figure S4: **Potential landscape and hBN valence-conduction bands in hBN/Au/Ni(111) and hBN/Ni(111) systems.** (a) We show the potential landscape for the hBN rectangular unit cell made of circles of radii d_N and d_B enclosing equal potentials V_{in} while the outside potential is set to V_{out}, where $\Delta(V_{out}\text{-}V_{in}) = 24.4$ eV. (b) Calculated band structures for the freestanding hBN/Au/Ni(111) and strongly interacting hBN/Ni(111) systems. (c) hBN valence band measured with ARPES (right) and simulated with the EPWE+PE model (left) for lattice-matched hBN/Au/Ni(111) (top) and hBN/Ni(111) (bottom) interfaces. ARPES data have been reproduced from Ref. 3. (d) Close-up view for the simulated dispersions highlighting the full hBN gap and its direct character for the two systems.

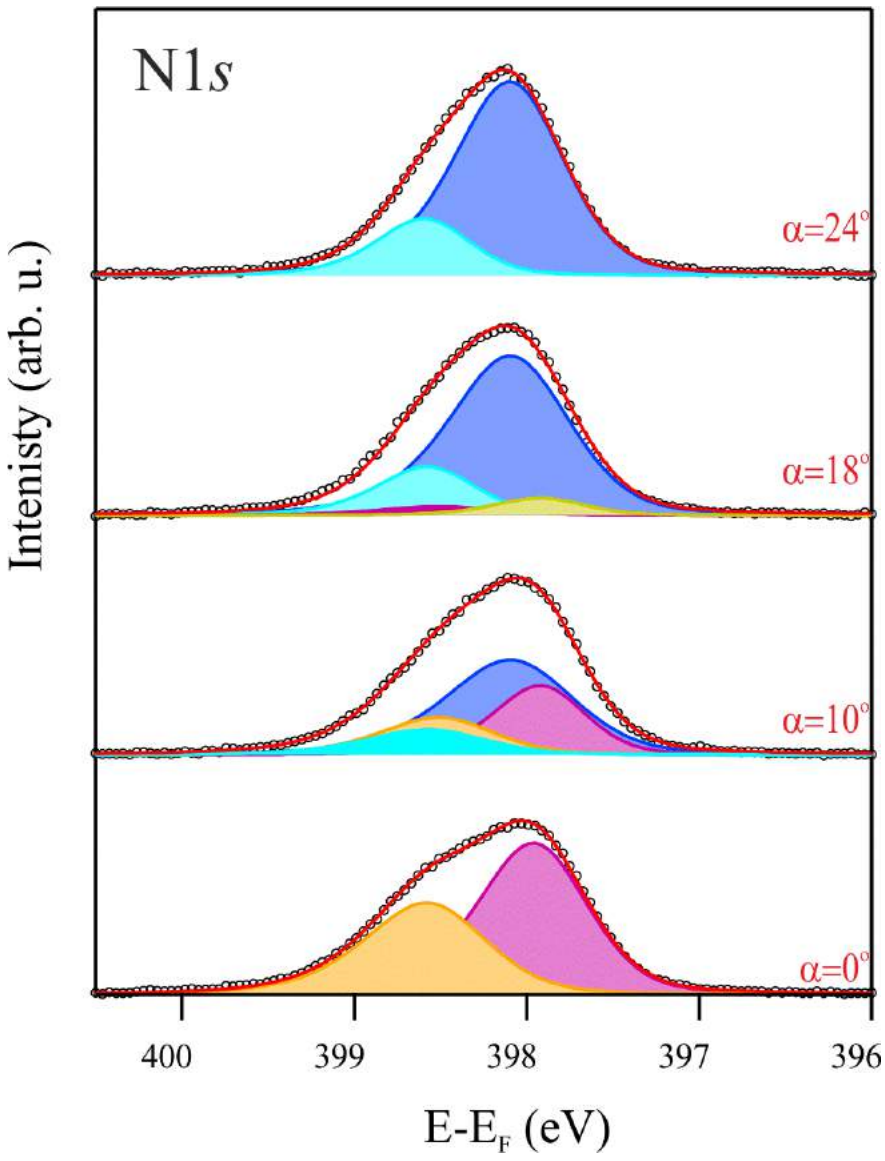

Figure S5: **X-ray Photoemission (XPS).** XPS N 1s core-level spectra at several vicinal angles across the hBN/c-Rh sample (hν = 480eV). Faceting is reflected in the smooth evolution from the pure (111) *nanomesh* phase at $\alpha = 0°$ to the hBN/Rh(337) interface at $\alpha = 24°$. Spectra at both points have been fitted with a double line for strongly interacting (pore and step, high binding energy) and less-interacting (wire, low binding energy) N atoms. For the fitting the intensity ratio of the pair of lines in each case has been fixed to the ratio of N atoms in pore/wire (0.4/0.6) step/non-step positions (0.2/0.8), respectively. In the (337) case, the shift in the lower-binding energy peak agrees with a $\sim$0.2 eV reduction in work function.

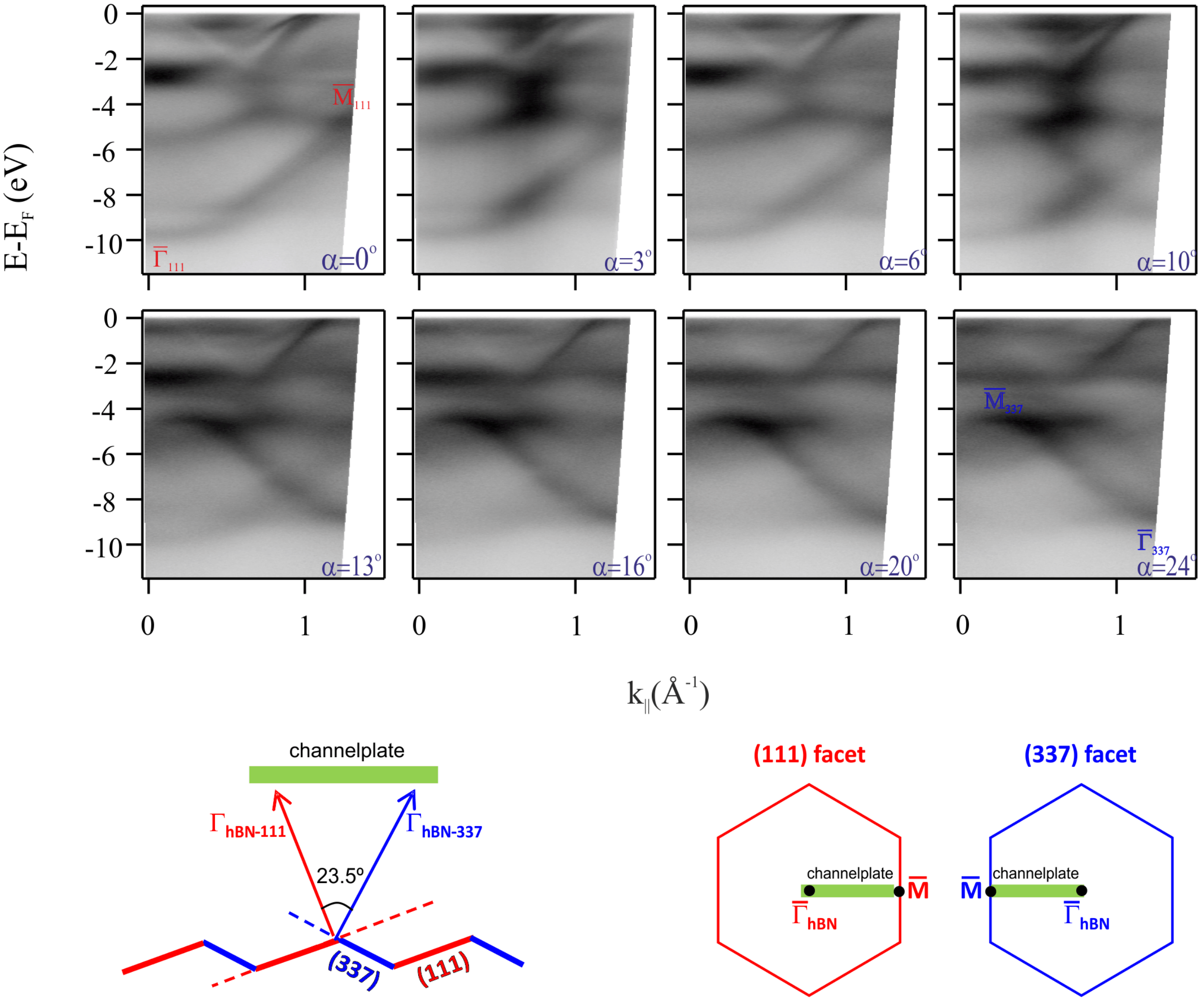

Figure S6: **ARPES from faceted structures.** Top, electronic bands of faceted structures across the hBN/c-Rh system, measured with ARPES along the $\bar{\Gamma}\bar{M}$ symmetry direction of hBN. Bottom-left, schematic description of the emission geometry from a faceted structure, with the normal emission angle located in between the two different emission planes. The tilted geometry ($\alpha = 23.5°$) of the facet planes leads to separate bands from each of the phases.[5,6] Bottom-right, the photon energy hν= 72 eV, is sufficient to scan the whole $\bar{\Gamma}\bar{M}$ line in both hBN-(111) and hBN-(337) phases.

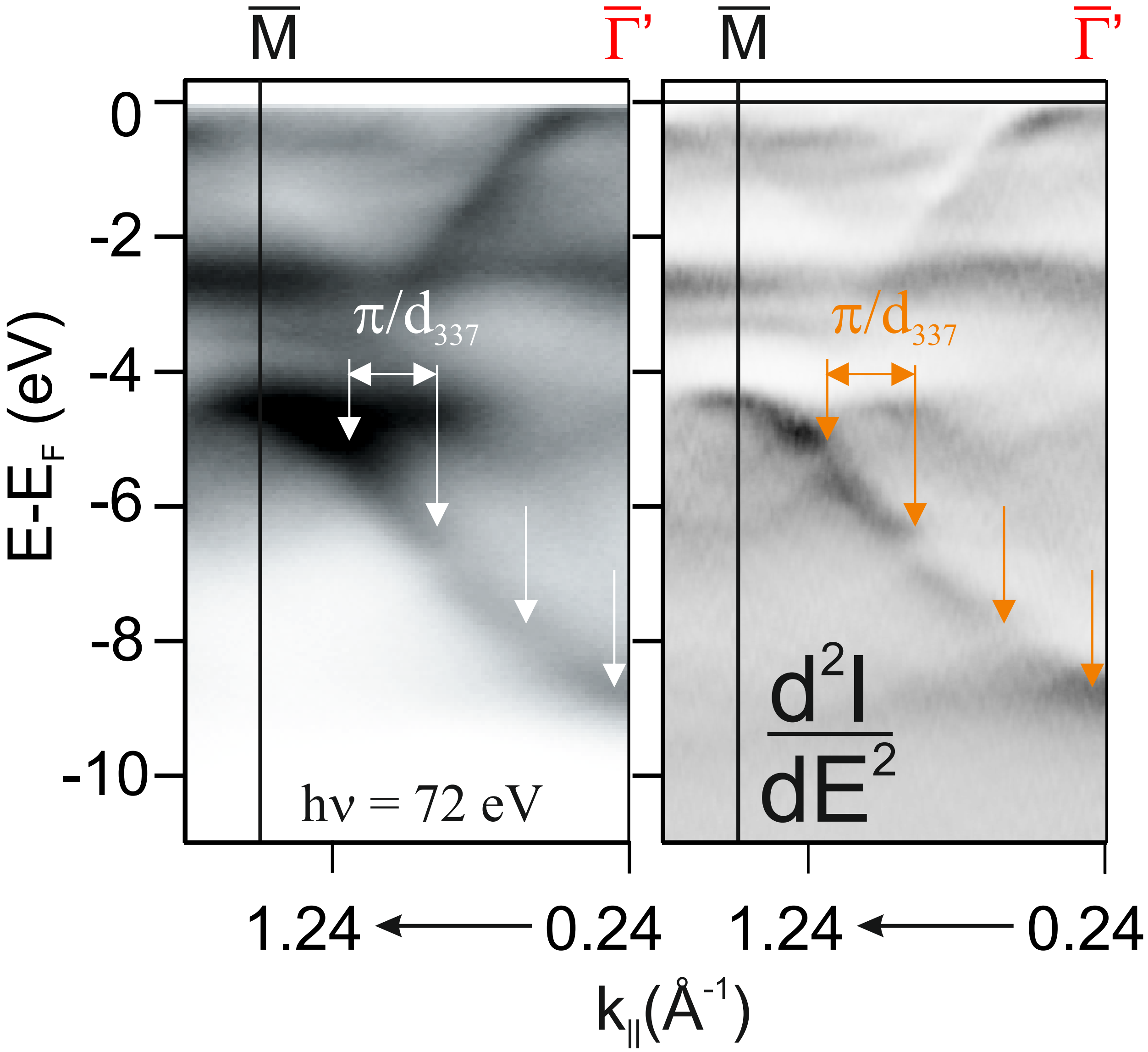

Figure S7: **Electronic bands of hBN/Rh(337).** Left, direct photoemission intensity measured with ARPES along the $\bar{\Gamma}'\bar{M}$ symmetry line of hBN, and at the Rh(337) point on the hBN/c-Rh surface. Right, same electronic bands displayed as second derivative of the photoemission intensity to enhance the observation of the different gaps. These are indicated by arrows. The $\bar{\Gamma}'$ point is defined in Fig. 4 of the main text.

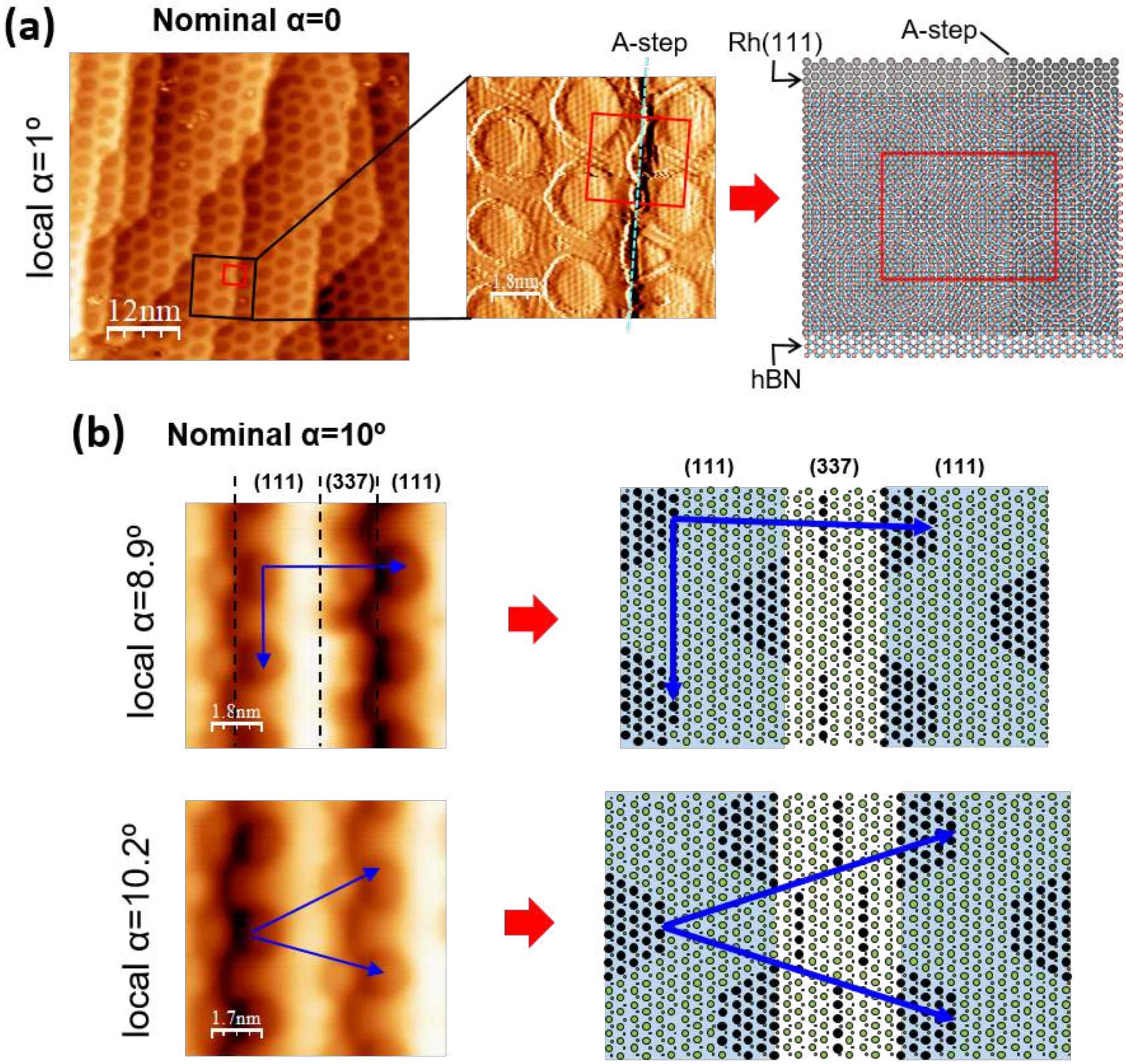

Figure S8: **Moiré modulation near monatomic steps and (111)/(337) interfaces.** (a) Atomically resolved STM images (left, topography; center, derivative) acquired near the (111) direction of the hBN/c-Rh system, showing the hBN *nanomesh* at a stepped patch (local 1° miscut). As shown in the right coincidence-lattice sketch, the square arrangement (red rectangle) proves the continuity of the hBN coating across the step. (b) STM image zoom-ins (derivative) extracted from two different points in the $\alpha = 10°$ image of Fig. 3 (a). Assuming a continuous hBN monolayer coat, the changing moiré arrangement across the (111)/(337) interfaces (blue arrows) are explained (right side) with the addition of a single (113) sub-unit (one monatomic step) in the Rh(337) substrate. Such fluctuation varies the local miscut by $\Delta\alpha = 1°$, that is, within the natural variation expected for a vicinal plane at $\alpha = 10°$.[7]

References

1. Abd El-Fattah, Z. M.; Kher-Elden, M. A.; Piquero-Zulaica, I.; de Abajo, F. J. G.; Ortega, J. E. Graphene: Free electron scattering within an inverted honeycomb lattice. *Phys. Rev. B* **2019**, *99*, 115443.

2. Kher-Elden, M. A.; Piquero-Zulaica, I.; Abd El-Aziz, K. M.; Ortega, J. E.; Abd El-Fattah, Z. M. Metallic bands in chevron-type polyacenes. *RSC Adv.* **2020**, *10*, 33844–33850.

3. Usachov, D.; Adamchuk, V. K.; Haberer, D.; Grüneis, A.; Sachdev, H.; Preobrajenski, A. B.; Laubschat, C.; Vyalikh, D. V. Quasifreestanding single-layer hexagonal boron nitride as a substrate for graphene synthesis. *Phys. Rev. B* **2010**, *82*, 075415.

4. Wickramaratne, D.; Weston, L.; Van de Walle, C. G. Monolayer to Bulk Properties of Hexagonal Boron Nitride. *The Journal of Physical Chemistry C* **2018**, *122*, 25524–25529.

5. Fernandez, L.; Makarova, A. A.; Laubschat, C.; Vyalikh, D. V.; Usachov, D. Y.; Ortega, J. E.; Schiller, F. Boron nitride monolayer growth on vicinal Ni(1 1 1) surfaces systematically studied with a curved crystal. *2D Materials* **2019**, *6*, 025013.

6. Mugarza, A.; Schiller, F.; Kuntze, J.; Cordón, J.; Ruiz-Osés, M.; Ortega, J. E. Modelling nanostructures with vicinal surfaces. *J. Phys. Cond. Mat.* **2006**, *18*, S27.

7. Ortega, J. E.; Vasseur, G.; Piquero-Zulaica, I.; Matencio, S.; Valbuena, M. A.; Rault, J. E.; Schiller, F.; Corso, M.; Mugarza, A.; Lobo-Checa, J. Structure and electronic states of vicinal Ag(111) surfaces with densely kinked steps. *New Journal of Physics* **2018**, *20*, 073010.
